# Programmed Cell Death Not as Sledgehammer but as Chisel: Apoptosis in Normal and Abnormal Craniofacial Patterning and Development

**DOI:** 10.3389/fcell.2021.717404

**Published:** 2021-10-08

**Authors:** Claudia Compagnucci, Kira Martinus, John Griffin, Michael J. Depew

**Affiliations:** ^1^Institute for Cell and Neurobiology, Center for Anatomy, Charité Universitätsmedizin Berlin, CCO, Berlin, Germany; ^2^Genetics and Rare Diseases Research Division, Ospedale Pediatrico Bambino Gesù, Rome, Italy; ^3^Department of Craniofacial Development, King’s College London, London, United Kingdom; ^4^School of Biological Sciences, University of East Anglia, Norwich, United Kingdom

**Keywords:** craniofacial, hinge and caps, *Satb2*, *Fgf8*, *Foxg1*, *Pbx*, branchial arch, apoptosis

## Abstract

Coordination of craniofacial development involves an complex, intricate, genetically controlled and tightly regulated spatiotemporal series of reciprocal inductive and responsive interactions among the embryonic cephalic epithelia (both endodermal and ectodermal) and the cephalic mesenchyme — particularly the cranial neural crest (CNC). The coordinated regulation of these interactions is critical both ontogenetically and evolutionarily, and the clinical importance and mechanistic sensitivity to perturbation of this developmental system is reflected by the fact that one-third of all human congenital malformations affect the head and face. Here, we focus on one element of this elaborate process, apoptotic cell death, and its role in normal and abnormal craniofacial development. We highlight four themes in the temporospatial elaboration of craniofacial apoptosis during development, namely its occurrence at (1) positions of epithelial-epithelial apposition, (2) within intra-epithelial morphogenesis, (3) during epithelial compartmentalization, and (4) with CNC metameric organization. Using the genetic perturbation of *Satb2*, *Pbx1/2*, *Fgf8*, and *Foxg1* as exemplars, we examine the role of apoptosis in the elaboration of jaw modules, the evolution and elaboration of the lambdoidal junction, the developmental integration at the mandibular arch hinge, and the control of upper jaw identity, patterning and development. Lastly, we posit that apoptosis uniquely acts during craniofacial development to control patterning cues emanating from core organizing centres.

## Introduction

Craniogenesis, the development of a patterned and functionally integrated head, is an especially elaborate process, one under intense developmental and evolutionary pressures (Gans and Northcutt, 1983; Depew and Simpson, 2006; Green et al., 2015; Marcucio et al., 2015). This applies to the pattern, development and morphogenesis of both the central nervous and the associated cranial peripheral nervous systems (CNS and PNS, respectively) as well as to the craniofacial tissues informing and supporting them, (i.e., to both the contents and the container). The developmental system that coordinates and patterns the craniofacial primordia involves a complex, intricate, genetically controlled, and tightly regulated spatiotemporal series of reciprocal inductive and responsive interactions. These include interactions between the embryonic cephalic epithelia (both endodermal and ectodermal) and the cephalic mesenchyme — particularly the cranial neural crest (CNC). The coordinated regulation of these interactions is critical both ontogenetically and evolutionarily, and the clinical importance and mechanistic sensitivity to perturbation of this developmental system is reflected by the fact that one-third of all human congenital malformations affect the head and face (Gorlin et al., 1990). Orofacial clefting alone (including cleft palate (CP) and/or cleft lip (CL)) constitutes, for example, one of the most common human birth defects (from 1 in 500 to 1 in 2,000 births depending on the population; Dixon et al., 2011; Ferretti et al., 2011).

Here, we focus on one element of this elaborate process — apoptotic programmed cell death (PCD) — and its role in normal and abnormal craniofacial development (CFD). Rather than intending an exhaustive catalogue, our aims are rather more modest. Firstly, we aim to provide a sufficient correlative overview of the mechanics and normal topography of apoptosis during CFD to promote the notion of apoptosis as an active biologic tool during the elaboration of CFD and morphogenesis, one acting more as a scalpel than as a sledgehammer. In this context, we present basic primers of both embryonic CFD (specifically highlighting early jaw development as an illustrative focal point) and the mechanics of apoptosis that together provide reference points as we discuss apoptosis in normal and abnormal CFD. These sections are intended to inform those new to the topics and can easily be skipped by those previously well-versed. We then describe features of the normal topography of apoptosis during early CFD, focusing on: (1) positions of embryonic epithelial-epithelial apposition and fusion; (2) intra-epithelial morphogenesis; (3) embryonic epithelial compartmentalization; and (4) early CNC organization. In addressing apoptosis in abnormal CFD we take two approaches. First, we turn to the molecular mechanics of apoptosis to underscore how CFD is (and is not) affected by mutations in the genes encoding components of the apoptosis machinery. In the other, we utilize examples from our own work that we believe exemplify the correlations between targeted mutations of craniofacial patterning genes (including of *Satb2*, *Pbx*, *Fgf8*, and *Foxg1*), aberrant CFD and morphogenesis, and the alteration of apoptotic profiles during CFD. And secondly, we utilize what has been presented from our first aim to posit a potentially unique role for developmentally programmed apoptosis in idiosyncratic aspects of early CDF and patterning.

### Craniofacial Patterning, Development, and Morphogenesis: A Brief Primer

**FIGURE 1:**
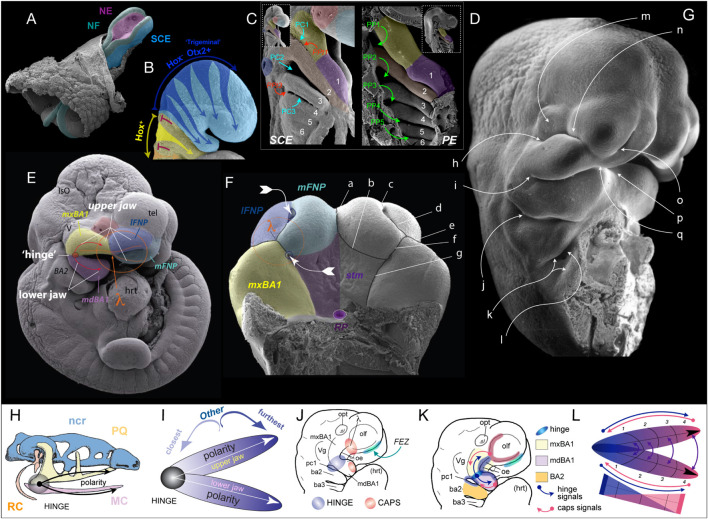
Early craniofacial patterning and development. **(A–G)** Scanning electron micrographs of shark **(A,C,D)** and mouse **(B,E–G)** embryos. **(A)** Nuerulation in a shark. NF, neural fold; NE, neurectoderm; SCE, surface cephalic ectoderm. **(B)** Fungible, trigeminal cranial neural crest (CNC) fills BA1 and the FNP. E9.25 mouse embryo. *Hox*^–^: blue. *Hox*^+^: yellow arrows entering BA2 from R4, orange entering BA3 from R6. Red T-bars indicate the apoptotic culling of R3 and R5 CNC. **(C,D)** SCE and PE in the BA of a hemisected shark embryo. 1–6, BA number. PC1–3, pharyngeal clefts 1–3 (SCE). PP1–5, pharyngeal pouches 1–5 (PE). PPt1/3, pharyngeal plates. lavender, mdBA1; yellow, mxBA1; red, optic primordia; light blue, FNP; dark blue, otic pit; orange, BA2. **(E)** E10.5 mouse embryo showing the topography of the craniofacial primordia, the hinge region and λ (the lambdoidal junction). IsO, isthmic organizer; tel, telencephalon; hrt, heart; lFNP, lateral frontonasal process; mFNP, medial frontonasal process; V, trigeminal ganglion. Colour as above. **(F)** E11.5 mouse embryo (with the lower jaw primordia removed) showing the embryonic primordia of the λ and correlated anatomical boundaries. The converging white arrows indicate the invaginating external nares and internal (primary) choanae whose infoldings must unite and perforate to form a nasal cavity (fossa) that communicates directly with the lower respiratory system via a nasopharynx. ***a***, internasal line (frontal groove); ***b***, naso-stomodeal line; ***c***, dorsal intranasal line; ***d***, ventral intranasal line (nasal fin); ***e***, nasolacrimal line (groove); ***f***, oronasal groove (medial-choanal line); ***g***, stomodeo-palatal line. **(G)** E11.5 mouse embryo highlighting positions of epithelial-epithelial apposition. ***h***, ridge of optical furrow from which the lower eyelid will sprout; ***i***, mxBA1-mdBA1 fusion at the oral commissure; ***j***, first pharyngeal cleft and plate; ***k***, opercular ridge of BA2 and epipericardial ridge with which it fuses; ***l***, cervical sinus; ***m***, recently pinched off lens; ***n***, nasolacrimal groove; ***o***, nasal fin; ***p***, mentum (mdBa1-mdBA1); ***q***, mFNP-mxBA1. **(H)** Schematic of prototypical vertebrate chondrocranium and the inherent polarity of it jaws. ncr, neurocranium; RC, Reichert’s cartilage; HINGE, circle at articulation; MC, Meckel’s cartilage; PQ, palatoquadrate cartilage. **(I)** Schema of the inherent polarity of appositional jaws. **(J)** Orientation of hinge and caps topography on an E10 mouse embryo. opt, optic primordia; Vg, trigeminal ganglion; FEZ, facial (frontonasal) ectodermal zone which helps integrate the frontonasal midline; oe, oral ectoderm. **(K)** Topography of epithelial signaling from the hinge (blue) and caps (red) signaling centres. **(L)** Modularity due to polarity in jaw development. Apposing gradients of signaling information create modules (here, 1–4) within the developing jaw primordia.

Much of our understanding of CDF follows from appreciation of the biology of, and patterning influences on, the CNC (Gans and Northcutt, 1983; Depew and Simpson, 2006; Bronner-Fraser, 2008); this brief primer focuses on this subject. Vertebrate craniogenesis typically begins in earnest with the advent of the recently gastrulated anterior mesendoderm coming to underlie the embryonic ectoderm and the initial partition of this ectoderm into neurectoderm (NE), surface cephalic ectoderm (SCE), and a transitional zone separating them (Figure 1A; Rubenstein et al., 1998; Depew and Compagnucci, 2008). While patterned regionalization progresses within the NE, CNC are eventually induced at the neural plate border lateral to the NE (Gans and Northcutt, 1983; Redies and Puelles, 2001; Bronner and LeDouarin, 2012; Green et al., 2015). Once induced and committed, CNC undergo an epithelial-mesenchymal transition (EMT) at the neural folds (NF) and migrate to colonize the regions surrounding the developing anlage of the ear, nose, eye, anterior CNS, and the meristic, metameric branchial (pharyngeal or visceral) arches (BA) (Arey, 1934; Nelsen, 1953; Goodrich, 1958; Wake, 1979; Minoux and Rijli, 2010; Etchevers et al., 2019; Figure 1B). The arches are transient embryonic structures of the developing head and neck for which an historic cacophony of naming schemes, depending upon anatomic tradition or phyla being described, exists (Figure 1C; Depew et al., 2005). To maintain continuity of naming with much of the literature being cited below we herein refer to the arches numerically from cranial to caudal as BA1 to BA6. The BAs will eventually give rise to many significant craniofacial structures, including most of the jaw skeleton (BA1), the auditory ossicles (BA1 and BA2), the muscles of mastication (BA1) and facial expression (BA2), the hyoid apparatus (BA2/3), the aortic arches, and their derivatives (e.g., portions of the aorta, truncus pulmonalis, and the carotid arteries; BA3/4/6), as well as the pharyngeal pouch (PP) tissues and glands (e.g., palatine tonsils, thymus, parathyroid, ultimobranchial body, and thyroid).

Significantly, cranio-caudal regionalization of the delaminating CNC initially establishes two disparate, functionally pre-patterned populations. One is an anterior equipotent *Hox*-gene negative, mostly *Otx2-*positive, population (aka., the “trigeminal” CNC) that migrates into BA1, surrounds the forebrain and eye, and migrates to colonize the frontonasal region (Figure 1B; Creuzet et al., 2002; Minoux and Rijli, 2010). It is this population that will directly yield the skeletal tissues of the jaws and anterior neurocranium. This CNC population is functionally interchangeable and neuro-axial origin does not direct disparate interpretations of subsequent positional patterning information. The second is a posterior *Hox*-gene positive population that fills the remainder of the BAs. Unlike the trigeminal CNC, the neuro-axial level of origin of this population does instruct the subsequent interpretation of positional information.

Being fungible in nature, the *Hox*-negative CNC receive positional patterning information locally from the cephalic epithelia. Thus, understanding the proximate patterning of the SCE and pharyngeal (foregut) endoderm (PE) is crucial to overall understanding of CFD. While the CNC is migrating from the NF, a signaling cascade establishes an iterative, cranio-caudal sequence of invaginations within the nascent PE that creates the pharyngeal pouches (PP; Shone and Graham, 2014). These invaginations then segregate the streams of CNC in the BAs by apposing and contacting corresponding pharyngeal (branchial) cleft (PC) invaginations of the SCE covering the BAs (Figures 1C,D). This contact transiently creates points of epithelial-epithelial apposition, each referred to as a pharyngeal plate (PPt). The epithelia of these contact points express signaling molecules that contribute to the patterning of the BAs (Depew and Compagnucci, 2008). Though the SCE naturally contains cells fated to be epidermal in nature, it also yields the cranial placodes (Pipsa and Thesleff, 2003; Bhattacharyya et al., 2004; Bailey et al., 2006; Patthey et al., 2014; Streit, 2018) and, notably, the known patterning centres for the anterior cranium and jaws (Depew et al., 2002a, b; Hu et al., 2003; Creuzet et al., 2006; Depew and Compagnucci, 2008; Minoux and Rijli, 2010; Cajal et al., 2014; Marcucio et al., 2015). To enable greater understanding of our subsequent exemplars of apoptosis in the context of normal and abnormal CFD it is expedient below to provide here some further detail of these patterning centres, BA1, and jaw development.

In part because the trigeminal CNC are, initially at least, equipotent in their response to local patterning cues, among the fundamental tasks in patterning BA1 and the jaws are: (1) establishing upper jaw versus lower jaw positional identity within the CNC, (2) generating and maintaining the point of articulation between the upper and lower jaw arcades, and (3) keeping the arcades in appropriate functional registration during subsequent development and morphogenesis. Actualization of these tasks involves elaboration of a “hinge and caps” system of establishing positional information in the CNC through several regional signaling centres (Depew et al., 2002a; Depew and Compagnucci, 2008; Compagnucci et al., 2013). BA1 is molecularly and developmentally subdivided at its “hinge” region into a distal, lower jaw-generating mandibular branch (mdBA1) and a more proximal, upper jaw generating maxillary branch (mxBA1; Figure 1E). Epithelially secreted signals from PPt1 (i.e., from PC1 and PP1, Figures 1C–E), coordinated with those from the oral ectoderm (OE; Figures 1J,K), generate a craniofacial organizing centre at the hinge that establishes the point of articulation (Rijli et al., 1993; Beverdam et al., 2002; Depew et al., 2002a; Depew and Compagnucci, 2008). Through mechanisms not yet fully understood, but clearly involving the restriction of *Dlx5/6* expression to mdBA1 (in part through *Foxg1* expression in the SCE and the regulation of Endothelin signalling in mxBA1) the hinge organizing centre likewise plays a significant role in instructing the CNC to either adopt a mxBA1-upper jaw identity or a mdBA1-lower jaw identity (Depew and Compagnucci, 2008; Sato et al., 2008; Compagnucci and Depew, submitted).

Crucially, BA1 primordia and the jaws that they give rise to are both fundamentally polarized (Figures 1H–L). This characteristic enables the functional registration of the upper and lower jaws from the point of their proximal articulations (at the hinge) to their distal endpoints (aka, their “caps”), and the hinge organizing centre alone is (theoretically) sufficient to generate such polarity. Notably, however, during gnathostome (jawed vertebrate) development, the upper jaws in all but Chondrichthyans incorporate a premaxillary component associated with the olfactory placode and capsule. Olfactory placodogenesis results in the invagination of a nasal pit into the underlying CNC, a morphogenic event that in amniotes distinctly delineates medial (mFNP) and lateral (lFNP) FNP primordia (Figures 1E,F). A significant transient embryonic structure of amniotes is the lambdoidal junction (hereafter λ) that develops where mxBA1, the lFNP and the mFNP converge (being fully elaborated around E10.5 in a mouse; Tamarin and Boyde, 1977; Depew et al., 2002a, b; Depew and Simpson, 2006; Depew and Compagnucci, 2008; Ferretti et al., 2011). In a topographically complex manner, the SCE of λ secretes a significant range of signaling molecules such that λ acts as the upper jaw “cap” organizing centre integrating mxBA1 development distal to the hinge region with that of the FNP. This includes the structural integration of the premaxillae, maxillae, and nasal capsules. It also includes the formation of choanae, unified lips, primary palates, and (in gnathostomes that possess them) secondary palates. As we will describe below, apoptosis plays a critical, clinically significant role in CFD associated with λ.

Though much less complex structurally and developmentally, the SCE of the distal-most midline of mdBA1 also secretes many of the same signaling molecules as λ — thereby creating the corresponding lower-jaw “cap” organizing centre. In sum, the CNC are patterned by the integration of the hinge organizing signals with countering epithelially derived patterning signals posteroproximally propagated through mdBA1 from the mandibular organizing “cap” and patterning signals posterodistally emanating through mxBA1 from the maxillary “cap” centred at λ (Figure 1K).

### Apoptosis: A General Description

Apoptosis, recognized as a specific subcategory of PCD, is a fundamental component of both animal development and adult homeostasis (Fuchs and Steller, 2011; Suzanne and Steller, 2013). Among the developmental mechanisms necessary for normal vertebrate morphogenesis are (1) epithelial invagination and evagination, (2) closure of epithelial tubes and vesicles, (3) separation of tissue components, (4) migration of anlage and rudiments, (5) the conjoining of appositional anlage, (6) the regulation of tissue specific cell-population size, and (7) the removal of only transiently necessary (or vestigial) tissues. As we will discuss below, the ontogenetic topography of such processes often correlates with the temporospatial topography of apoptosis (Glücksmann, 1951; Saunders, 1966; Kerr et al., 1972; Jacobson et al., 1997; Fuchs and Steller, 2011; Ghose and Shaham, 2020; Voss and Strasser, 2020). As an evolutionarily conserved mechanism of organismal cellular auto-culling, apoptosis results in a controlled, distinct, canonical and readily recognizable set of changes in cellular ultrastructure. Such changes include: (1) the rounding and shrinking of cell size (cytoplasmic compaction); (2) nuclear changes accompanied by the condensation and marginalization of chromatin (pyknosis) and eventual internucleosomal DNA fragmentation (karyorrhexis); (3) organelle shrinkage, fragmentation and rearrangement of cytoskeletal elements; (4) the advent of plasma membrane blebbing and the eventual separation of cell fragments into apoptotic bodies; (5) the release of “Apoptotic cell-derived extracellular vesicles” (ApoEVs) and their subsequent specific communications with surrounding tissues; and (6) the eventual phagocytic engulfment and degradation of cellular corpses (Kerr et al., 1972; Voss and Strasser, 2020). While other forms of PCD and senescence have been characterized, as well as have pathological forms of cell death, we are herein primarily concerned with apoptotic PCD and its roles in craniofacial pattern, development, and morphogenesis.

### Overview of Historic Thoughts on Apoptosis’ Role in Development

Numerous posited rationales for why an organism would benefit from programming intra-organismal cellular death have been put forth. Appreciation of plausible roles for PCD during embryonic development has historically revolved around differentially perceived developmental and morphologic needs to eliminate *unwanted* cells, in particular during such developmental and morphogenic processes topographically correlated with apoptosis as those briefly enumerated above (e.g., epithelial invagination; Glücksmann, 1951; Saunders, 1966; Kerr et al., 1972; Shuler, 1995; Jacobson et al., 1997; Meier et al., 2000; Fuchs and Steller, 2011; Voss and Strasser, 2020). Briefly amalgamating posited developmental roles here, apoptosis has, for instance, been associated with the elimination of cells that seem to have no function as they are part of the development of a vestigial structure. This is exemplified by the programmed loss of mammalian pronephric tubules (exemplifying “phylogenetic” apoptosis) and the sex-specific reduction of either Müllerian or Wolffian ducts (“histogenic” apoptosis) (Glücksmann, 1951; Saunders, 1966; Jacobson et al., 1997). Apoptosis is likewise a perceived developmental mechanism that eliminates cells that have already functioned during embryonic ontogeny but which are no longer necessary. The elimination of Cajal-Retzius cells, the seminal colonizer neurons of the outer layer of the developing neocortex that are essential for both establishing the initial neocortical layering and functionally interacting with the overlying nascent meninges, exemplifies this type of role (Wong and Marín, 2019). Moreover, tissue specific ontogeny, such as with cerebral cortical development, can often be an intricate dance between progenitor cell division and proliferation on the one hand and regulated cell-type specific differentiation on the other. This dance often includes steps leading to the generation of excess numbers of cells, and apoptotois has been well documented as a significant mechanism for subsequently controlling appropriate neuronal cell numbers during development (Vecino and Acera, 2015; Wong and Marín, 2019). Apoptotic function has also been ascribed to the ontogenetic elimination of cells that are outright abnormal, misplaced, non-functional, or harmful, including limiting T- and B-cell lymphocyte expansion where reactivity to self or failure to form useful, antigen-specific receptors is at play.

These examples of larger scale developmental apoptosis are representative more of a sledgehammer-like rather than of a scalpel-like approach to cellular elimination. A notable scalpel-like corollary here comes when the population of cells under size control is small and contains cells actively secreting morphogen patterning signals. This is seen in the regulative cell death zone associated with controlling the numbers of Shh*-*expressing cell in the zone of polarizing activity of the limb bud (Sanz-Ezquerro and Tickle, 2000), the anterior neural ridge (Nonomura et al., 2013), and in the enamel knot signalling centres of developing teeth (Vaahtokari et al., 1996; Ahtiainen et al., 2016). Importantly, “morphogenic” apoptosis has also been deemed a principal sculptor of embryonic structures during morphogenesis, organogenesis and remodelling - the holotype sculpture being the apoptotic elimination of cells in the inter-digital webbing of the developing amniote autopod (Glücksmann, 1951; Saunders, 1966; Hinchliffe, 1981).

### Molecular Toolbox and Machinery Underlying Apoptois

With this cursory look at the “where” and “why” of apoptosis in development, we briefly consider “how” it occurs. The molecular mechanisms underlying apoptosis have been well reviewed elsewhere (e.g., Jacobson et al., 1997; Meier et al., 2000; Taylor et al., 2008; Fuchs and Steller, 2011; Ravichandran, 2011; Tuzlak et al., 2016; Dorstyn et al., 2018; Voss and Strasser, 2020; Wanner et al., 2021) and are thus only succinctly presented here. Depending on the source of apoptotic instigation, two convergent mechanistic pathways of apoptosis - an intrinsic (or mitochondrial) and an extrinsic (or death receptor) - have typically been recognized. The intrinsic pathway is developmentally initiated by such events as a local change in growth factor or neurotrophic support level, metabolic stress, or extracellular matrix (ECM) detachment and epithelial cell extrusion (i.e., forms of anoikis). Much like many other molecular and cellular equilibrating acts, the intrinsic pathway response to apoptotic stimuli is believed to be regulated by a balanced activity of two classes (i.e., BH3-only and multi-BH domain) of pro-apoptotic members of the BCL-2 gene family with the pro-survival (anti-apoptotic) BCL-2 members (e.g., BCL-2, BCL-W, BCL-XL, A1/BFL-1, and MCL-1) which converge on caspases (cysteine-aspartic proteases) to effectuate apoptotic cell death. In the un-stimulated steady-state, anti-apoptotic BCL-2 proteins keep pro-apoptotic BH3-only effectors, such as BAK, BAX and BOK, in an inactive state. Once presented with apoptotic stimuli, however, BH3 proteins (e.g., BAD, BID, BIK, BIM, HRK, NOXA, and PUMA) inhibit anti-apoptotic BCL-2 proteins, thereby releasing effector BH3-only proteins to oligomerize and perforate the mitochondrial outer membrane (MOM) by pore formation. Once perforated, MOM permeabilization allows the egress of cytochrome c into the cytoplasm, wherein it interacts with APAF-1 (apoptosis activating factor 1) and an *initiator* caspase, such as procaspase-9, to create an “apoptosome” and sequently enzymatically activate the initiator procaspase. Activated Caspase-9, in turn, enzymatically cleaves a second class of inactive procaspases, the *executioner* (or effector) caspases, in particular Caspases-3, -6, and/or -7. This activates executioner caspases to cleave - and so to either activate or inhibit - a multitude of other proteins. Among these newly activated proteins are endonucleases that drive DNA fragmentation and inhibitors of flippases which then unbalance the constituent components of the bi-layered organization of the plasma membrane. Subsequently, these latter events reveal “find me” and “eat me” signals leading to the eventual phagocytic engulfment and degradation of apoptotic debris (Ellis et al., 1991; Taylor et al., 2008; Fuchs and Steller, 2011). Apoptotic stimuli, moreover, may also liberate initiator caspases from their inhibition by various IAP (Inhibitors of Apoptosis, such X-linked inhibitor of apoptosis, or XIAP) gene family encoded proteins.

As suggested by its sobriquet, the extrinsic death receptor pathway is initiated by the cognate receptor binding of tumour necrosis factor (TNF) family ligands, in particular of FAS ligand (FASL) to FAS receptor, which then potentiates two distinct series of events. When present, procaspase-8 is activated (through a TRADD-RIPK1-FADD pathway) to cleave and thereby activate effector Caspases-3, -6, and/or -7 which can effectuate the proteolytic conversion of inactive BID into activated tBID — which then activates BAK and BAX with predicable consequences. Absent facient Caspase-8, the necroptotic cell death regimen (involving RIPK1, RIPK3 kinase activation, and MLKL phosphorylation and membrane translocation) can be initiated; notably, however, this leads to changes in cellular morphology that are distinct from those encountered with apoptosis and the process is thought to rarely occur during development (Voss and Strasser, 2020).

Axiomatically, expression of the components of the apoptosis machinery are under the disparate regulation by numerous transcription factors, including p53/63/73, AP-1, NF-κB, IRF-1, Myc, Foxo, Hox, and Snail (Dudgeon et al., 2009). Expression of such factors collectively establishes a regulatory balance between pro-apoptotic and anti-apoptotic proteins in the cell and thus ultimately controls cell death decisions at the cellular level.

### Apoptosis as Chisel I: The Topography of Apoptosis During Normal CFD

In his compendium 70 years ago on cell death in normal vertebrate ontogeny, Glücksmann (1951) enumerated numerous accounts of cell death associated in some fashion with CFD. As with elsewhere in the vertebrate body, he established that cell death could often be correlated with craniofacial tissues changing shape. Among those topographies that he enumerated were the developing optic lens, vesicle and cup, the otocyst, the oronasal junction, the PPts, and the palatal shelves. To date, however, a comprehensive exposition of the normal, predictable spatiotemporal ontogeny of apoptosis explicitly in the craniofacial tissues of vertebrate embryos has yet to materialize. Sufficient subsequent efforts at detection of apoptotic PCD, typically through TdT-mediated dUTP nick end labeling (TUNEL), electron microscopy, or immunohistochemical assays of activated Caspase-3, permits some further categorization of the predictable time and place of the occurrence of embryonic craniofacial apoptosis. Though not intended to be exhaustive in nature, and noting occasional overlap between categories, we highlight four evolving themes in the temporospatial elaboration of apoptosis during CFD as occurring: (1) at positions of epithelial-epithelial apposition; (2) within intra-epithelial morphogenesis; (3) during epithelial compartmentalization; and (4) with CNC metameric organization. With most of these we suggest that apoptosis acts as a fine tool to sculpt the developing tissues for the needs of subsequent development and morphogenesis.

#### Apoptosis and Epithelial-Epithelial Apposition and Fusion

**FIGURE 2:**
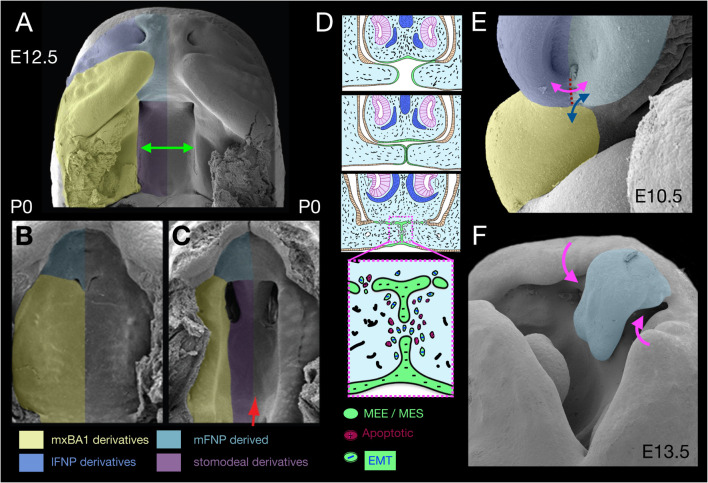
Apical-apical epithelial apposition, orofacial clefting, and apoptosis. **(A–C)** Pseudocoloured scanning electron micrographs of the secondary palate at E12.5 (**A**, green arrow) and P0 **(B)**. The secondary palate in panel **(C)** is cleft due to a *deficiency* of *Gas1*. **(D)** Schema of the apposition of nasal septum and secondary palatal shelves wherein apoptosis acts in conjunction with epithelial-mesenchymal transition (EMT) and migration to remove the MEE/MES. **(E)** λ in an E10.5 mouse embryo. Dotted red line, topography of nasal fin. Double headed arrows indicate significant appositions. magenta, nasal fin; blue, mxBA1-mFNP. **(F)** E13.5 mouse embryo evincing a cleft lip and cleft primary palate (magenta arrows). Blue psuedocolour indicates the mFNP derivative.

Craniofacial apoptosis is predictably and consistently found where cranial epithelia of one source grows to meet, in apposition, other epithelia — especially where the meeting, fusion, and subsequent degeneration is fundamental to further growth and development (Figures 1F,G). As we present below, such topographies are exemplified by the appositions of the developing palatal shelves, the meeting of the FNP and mxBA1 at λ, the eyelids, the PPts and precervical sinus, and the buccopharyngeal membrane. Indeed, defects in epithelial fusion in any such local typically result in the development of clinically important congenital malformations (Ray and Niswander, 2012; Molè et al., 2020). This apposition may be apical-apical in nature wherein contact is initiated at the apical surfaces of the two epithelia, a situation especially common where it involves two SCE-derived epithelia (Figures 1G, 2). Such an apposition is typified, for example, by the apposition of the mammalian secondary palatal shelves, where they merge with the primary palate, and where the nasal septum joins the palate (Shuler, 1995; Cuervo and Covarrubias, 2004; Jin and Ding, 2006; Yamamoto et al., 2020). Apoptosis-associated apposition may also be basal-basal in nature wherein initial contact between epithelial sheets occurs along their basal lamina and basement membranes. This is typified by the apposition of the PE and the SCE, such as at the buccopharyngeal membrane and the PPts (Watermann, 1977; Shone and Graham, 2014; Chen et al., 2017).

Typical of apical-apical apoptotic appositions is the growth and convergence of the mammalian secondary palate, a multi-step process that begins with specification of CNC within mxBA1 (Dixon et al., 2011). This is followed first by a vertical mesenchymal growth phase where the shelves initially expand vertically from mxBA1 along the sides of the growing tongue and then, subsequently, by reorienting the direction of proliferation and extending horizontally above the tongue and towards the midline (Figure 2A). During these phases an essential, specialized superficial layer of the epithelia, the periderm, covers the developing palate, acting as a barrier to unwanted epithelial-epithelial adhesions (e.g., with the tongue) that would mechanically arrest palatal growth and fusion and result in CP (Richardson et al., 2014). Horizontal growth continues until the contralateral shelf epithelia, here commonly referred to as medial edge epithelium (MEE), make contact and fuse to generate a midline epithelial seam (MES; Figure 2D). In order for a continuous palatal shelf to form, and thereby separate the oral and nasal cavities, the cells, basal lamina and basement membranes of the MEE must be removed at the MES. This allows palatal fusion at the midline and failure to achieve this leads to CP. After years of significant contention over the primacy of cellular mechanisms of MES disintegration, genetic fate mapping and manipulation in mice suggests three mechanisms are each involved: (1) apoptosis, (2) EMT, and (3) epithelial cell migration and extrusion either towards the oral or nasal palatal surface (Cuervo and Covarrubias, 2004; Losa et al., 2018).

Notably, apical-apical apoptotic apposition is also seen at λ where the epithelium of the inferolateral mFNP converges (beginning around E10.5 in a mouse) to meet mxBA1 and the inferolateral lFNP at the ventral intranasal line (nasal fin; Figures 1F,G, 2). As with the palatal shelves, apposing epithelial cells first appear to extend fillipodia across the gap separating both halves in order to intercalate and anchor themselves in between cells of the contralateral anlage. This then facilitates the coalescence of the appositional epithelial sheets and generates an epithelial seam at the nasal fin (Cox, 2004; Jiang et al., 2006; Ray and Niswander, 2012). The seam must subsequently disintegrate in order for the full, normal mergence of the primordia at λ to result in the formation of the primary palate and a continuous, externally even upper lip. Failure of this process leads to CL and primary CP (1CP; Figure 2F). Apoptotic culling helps to ensure that adequate cell numbers are removed from the tips of the epithelial seams to facilitate adhesion of the converging midfacial processes and occurs before and during mFNP, mxBA1 and lFNP fusion. Analysis of the levels of apoptotic cells of the disintegrating seam from E10.5 to E11.5 in mice (being highest around E10.75) has suggested that no more than 40% of the SCE-derived epithelial cells at λ undergo apoptosis, a percentage thought to be insufficient to the clearance of all the nasal fin cells (Losa et al., 2018). Thus, full nasal fin epithelial disintegration involves additional mechanisms. As with the palatal shelves, fate mapping and conditional genetic manipulation in mice suggests the same three general mechanisms that are involved in MES disintegration — that is, apoptosis, EMT, and epithelial cell migration — act in concert to bring about normal seam disintegration at λ (Losa et al., 2018). Critical to the overall process, apoptosis here is topographically highly focal and surgical (i.e., is scalpel-like).

Though best studied due to their greater clinical urgency, apical-apical apposition of the palatal shelves and at λ are certainly not the only places such appositions occur during craniofacial development. Apoptotic cells are evident where epithelia of mxBA1 and mdBA1 apically appose at the OE of the hinge (i.e., the embryonic oral commissure) and subsequently fuse to ensure the buccal organ is continuous laterally and the obicularis oris muscle can completely encircle the oral opening (Lotz et al., 2006). Failure of fusion along this part of the orotragal line underlies macrostomia (Kobraei et al., 2016). The mdBA1 midline (mentum) epithelia likewise undergo apoptotsis-positive apposition and their failure to do so is associated with lower median cleft lip (Oostrom et al., 1996; Lotz et al., 2006). Apoptosis is also detected in the epithelium of contralateral mFNP when they appose, and failure to fuse at the midline results in mid facial clefting (Griffin et al., 2013; Lantz et al., 2020). In each of these cases, apoptosis appears to cull relatively small numbers of cells to achieve a significant sequent morphological affect.

Formation, migration, closure and subsequent re-opening of the eyelids provides yet another example of complex craniofacial apical-apical epithelial appositions for which apoptosis appears as a part of the normal ontogenic progression. The optic and periocular SCE undergoes repeated rounds of coordinated epithelial morphogenesis and migration during development — to first generate a lens placode, and then to invaginate the placode to form a lens vesicle, pinch off completely as a vesicle from the SCE, generate a distinct corneal layer, build and properly position corneal (conjunctival) furrows, and then undergo focal glandular branching morphogenesis and tubulogenesis (to contribute, for example, to the formation of the nasolacrimal duct). Importantly, the SCE-derived epithelium must also establish a migratory wave of cells, encased in an epithelial periderm layer, to build eyelids that will grow as migratory waves across the sclera and then appose one another over the cornea, fuse and eventually split open again — all without becoming physically attached to the corneal or scleral surfaces (Pei and Rhodin, 1970, 1971; Harris and McLeod, 1982; Findlater et al., 1993; Meng et al., 2014). Once the upper and lower eyelid anlage converge, epithelial apposition creates an unique epithelial seam — the eyelid junction epithelium (EJE) — replete with cellular extrusions. However, unlike with the apical-apical fusions of the palate or the nasal fin, which need to disintegrate comparatively rapidly to keep up with palatal growth, the fusion of the eyelids must be maintained for a protracted period of time. This difference results in distinct cellular and extracellular dynamics relative to the palate or nasal fin (Ray and Niswander, 2012; Meng et al., 2014). However, once the proper developmental stage has been reached, and apoptotic signals (likely through Smad1 mediated death-receptor up regulation) are received, the EJE undergoes focal apoptosis (Sharov et al., 2003; Meng et al., 2014). This, combined with stimulated keratinocyte differentiation, is thought to lead to eyelid separation and opening. Significantly, the cephalic end of vertebrate embryos exhibits numerous other examples of apical-apical appositions and fusions apparently involving apoptosis. Many of these, such as neural tube closure (NTC), have been well documented and are amply discussed elsewhere (e.g., Yamaguchi and Miura, 2013; Nikolopoulou et al., 2017; Molè et al., 2020).

As noted above, basal-basal epithelial apoptosis-associated appositions likewise occur during normal craniofacial development, and includes both SCE-SCE and SCE-PE epithelial contacts. In addition to the primary and secondary palates and the upper lip, morphogenesis at λ includes the formation of the primary chonae, the internal oronasal aperture (Tamarin, 1982). Externally, as the amniote nasal pits invaginate between the mFNP and lFNP (to eventually create the nasal duct) each primary choanal pit begins its invagination at the medial end of the oronasal groove (medial-choanal line; Figure 1F). After significant intranasal epithelial morphogenesis, coupled with differential proliferation of the underlying CNC, an oronasal membrane composed of two SCE-derived epithelial sheets with apposing basement membranes separates the oral and nasal cavities (Tamarin, 1982). Rupture of this membrane is essential for the tetrapodal ability to breath with the mouth closed, proceeds without cell intercalation, and is accompanied by apoptotic cells at the site of disintegration (Weingaertner et al., 2006; Chen et al., 2017). Here, again, apoptosis appears to play a more surgical role than an obliterative one.

SCE-PE basal-basal epithelial apoptosis-associated appositions also characterize the development and rupture of both the buccopharyngeal membrane and the PPts (Watermann, 1977; Tamarin, 1982; Shone and Graham, 2014). The buccopharyngeal (oral) membrane separates the SCE of the stomodeum from the foregut PE, and its rupture is essential for vertebrate life. Rupture appears to follow stepwise beginning with SCE reorientation, controlled loss of basement membrane integrity, apoptotic culling and cell migration within the SCE, and eventual SCE-PE cellular intercalation followed by rupture. Absence of rupture, though rare, does occur. Of particular comparative evolutionary importance are the SCE-PE appositions. Accumulating evidence suggests that it is the reiterative pattern and proliferation within the forming PE that instigates PP-PC contact (Haworth et al., 2004; Graham et al., 2005). The behaviour of this contact differs cranio-caudally within the PPts (Shone and Graham, 2014). With the first PPt, once contact is initiated the PE epithelia regresses leaving an intact PC epithelia (which is necessary for the continuity of the tympanic membrane). For the caudal PPts, apoptosis is evident — mostly in the SCE-derived PC — allowing the PP epithelia to intercalate and displace the ectoderm whereupon the PPts either rupture or maintain their integrity while allowing the PE epithelia to extrude between the BAs (Shone and Graham, 2014). Subsequently, the caudal (aboral or “opercular”) border of BA2 typically undergoes a distinctive proliferation that results in its overlapping of the cervical sinus, a transient depression containing the remaining caudal BA (Figures 1G,K; Frazer, 1926). In tetrapods, this opercular border will eventually grow caudad to appose and fuse, along its caudal margin, with the epipericardial ridge. In doing so it must act internally with the now mixed SCE-PE epithelial populations of the lateral surfaces of the BA (Shone and Graham, 2014). Errant development of this process results in fistulae of the ear and neck (Arey, 1934).

#### Apoptosis and Intra-Epithelial Morphogenesis

Epithelial folding is a fundamental morphogenic event as it acts to transform simpler two-dimensional epithelial sheets into complex three dimensional structures that are often essential for tissue or organ function as well as for organismal life (Davidson, 2012). Indeed, much of vertebrate embryonic craniofacial morphogenesis and organogenesis is the product of forces acting within and on epithelial sheets. With respect to embryonic epithelial folding, this mechanistically includes changing forces initiated by apoptotic cells (Suzanne and Steller, 2013; Monier and Suzanne, 2015; Monier et al., 2015). For instance, some apoptotic epithelial cells have been shown to regulate epithelial folding by utilizing a dynamic pico-basal myosin II cable to exert a transient pulling force on the apical surface of the epithelium in which they are dying (Monier et al., 2015). Among the numerous examples of significant craniofacial tissues for which intra-epithelial morphogenesis involves apoptosis are the optic lens and mammalian teeth. Apoptosis is clearly found during lens pit formation and morphogenesis (see below) where it is believed to be necessary to establish the correct number of cells in the invaginating lens pit and to physically separate from the surrounding SCE (Findlater et al., 1993; Morgenbesser et al., 1994; Pan and Griep, 1995; Lang, 1997; Meng et al., 2014). The three dimensional cusp pattern of mammalian molars takes shape through the enamel knot signaling centres that are developmental cleared through apoptosis (Vaahtokari et al., 1996; Pipsa and Thesleff, 2003; Ahtiainen et al., 2016).

#### Apoptosis and Epithelial Compartmentalization

While the NE, PE and SCE each undergo compartmentalization, we selectively highlight here the specification, individuation and subsequent development of the cranial sensory placodes as exemplifying regionalization and compartmentalization that mechanistically involves apoptosis. From the presumptive SCE arises a pre-placodal region (PPR), unique to this surface ectoderm, that has been shown to represent a specialized competence for placodal induction (Jacobson, 1963; Washausen et al., 2005; Bailey and Streit, 2006; Schlosser, 2010; Saint-Jeannet and Moody, 2014; Streit, 2018). Experimental evidence supports the notion that once the PPR has been specified there follow sequent inductive steps to regionalize identity as posterior (otic and epibranchial) and anterior (olfactory, lens, and adenohypophyseal), with the trigeminal being intermediate. It is within the anterior PPR that the SCE will give rise to the epithelia of λ. Within this anterior domain all placodal progenitor cells are initially specified as lens regardless of subsequent fate, and induction of non-lens fate requires both the active induction of unique identity (through distinct sets of inductive cues) and the active repression of lens fate (which is mediated in part by the CNC; Bailey et al., 2006). Within all sensory placodes, moreover, individual neural placodal fates have to be actively acquired and cellular differentiation and delimitation controlled. The trigeminal and epibranchial placodes, for instance, will physically generate ganglionic neuroblasts that must delaminate and intermingle with CNC-derived cells to create (1) the trigeminal ganglia of cranial nerve (CN) V, (2) the geniculate ganglion of CNVII, (3) the petrosal ganglion of CNIX, and (4) the nodose ganglion of CNX. Meanwhile the olfactory and otic placodes will generate, *in situ*, their own specialized neurosensory cells. Strong evidence indicates that topographically regulated apoptosis normally seen within the amniote SCE assists the eventual individuation of the trigeminal and, within the PPR, the physical compartmentalization of the epibranchial and otic placodes (Washausen et al., 2005; Knabe et al., 2009; Washausen and Knabe, 2013). Notably, aquatic vertebrates have unique lateral line placodes derived from within the PPR that generate mechanosensory and electosensory neuroblasts well suited to life in water. It has recently been demonstrated that, in cultured whole murine embryos in which apoptosis is arrested by the pan-caspase inhibitor by Q-VD-Oph, lateral line placodes develop in the PPR topographically within the apoptotic domains that also help discriminate the epibranchial placodes from the otic placode (Washausen and Knabe, 2018). Thus, in an apparent example of Glückmann’s phylogenetic apoptosis, PCD within the amniote SCE removes the potential for unnecessary lateral line placodogenesis.

#### Apoptosis and CNC Organization

Much of the vertebrate embryo is segmental in nature. In post-cranial development, metameric organization (including of the truncal neural crest) follows mesodermal positional cues (Graham et al., 1996). The cranial ends of vertebrate embryos are also metameric, in particular the BAs and the rhombomeres (R) of the hindbrain (Graham et al., 1993; Minoux and Rijli, 2010). Fate mapping studies of the CNC has shown a correspondence of rhombomeric segmentation, neural crest emigration, and BA metamerism (Noden and Trainor, 2005). Anteriorly, the fungible trigeminal CNC emigrates to line BA1, surround the forebrain, and take position below the anterior SCE. This equipotent *Hox*^–^ population includes cells that delaminate from R1 and R2, the midbrain, and the posterior forebrain. The *Hox*^+^, post-trigeminal CNC of BA2 and BA3 mostly emigrates from the even numbered posterior R such that R4 CNC enters BA2 while R6 CNC enters BA3. This coordinated segmentation is key to generating positional information between the BAs. Notably, this mechanism for pattern control is actively maintained by the early apoptosis-induced elimination of CNC at the third and fifth rhombomeres (Graham et al., 1993).

### CFD in the Face of Mutations of the Apoptotic Machinery or Regulators of Its Transcription

Understanding of the mechanistic control of a great many developmental processes has enormously benefited from successful reverse genetics approaches in mice. Sometimes, however, such approaches muddy the waters of initial understanding, especially when expectation of phenotype fails to fit phenotypic outcome in loss of function studies. This appears to have been the general case with regard to many of the initial interpretations of the loss of function of components of the apoptotic machinery either as individual or as compound mutations (e.g., Linsten et al., 2000; Tuzlak et al., 2016). For instance, null mutations in *Apaf1* in mice resulted in significant rostral neural tube closure defects, mid-facial clefting, failure of palatal shelf fusion, and jaw dysmorphology (Cecconi et al., 1998; Yoshida et al., 1998; Long et al., 2013). These significant malformations set expectations relatively high that mutations in other components of the apoptotic machinery would yield at least similar defects. When null mutations for other machinery components, such as BAK and BAX (either singly or in compound), were reported to have more underwhelming phenotypic consequences (Linsten et al., 2000) these expectations were revaluated (Tuzlak et al., 2016). A survey of single and compound mutations of the individual components of the apoptosis machinery, however, indicates that abnormal craniogenesis - of both the contents and the container - is as consistent a recognized phenotype as any with the loss of function of the apoptosis machinery (e.g., Voss and Strasser, 2020). With regard to apoptosis during CFD, however, two factors have subsequently manifested. First, the apoptotic machinery appears to have built-in redundancies (as might be expected for a system in need of regulatory balance). This becomes evident, for example, with the compound mutations of like-type components of the apoptotic machinery such as the pro-apoptotic BH3-only effectors BAK, BAX, and BOK (Ke et al., 2013, 2018). In the absence of BOK alone no readily discernible phenotypic abnormalities are noted (Ke et al., 2013). With BOK homozygosity compounded with losses of either BAK or BAX, phenotypic abnormalities are only slight exacerbations of the small phenotypic changes noted with single null mutations of BAK or BAX (Ke et al., 2013). Compound mutation of all three, however, results in significant craniofacial defects, including exencephaly, midfacial clefting and hypognathism of the lower jaw (Ke et al., 2018). Compound mutations of the anti-apoptotic BCL-2 members MCL-1 and BCL-X likewise result in craniofacial defects including midline truncations of the upper and lower jaws (Grabow et al., 2018). Analysis of the loss of function of key transcriptional regulators of the apoptotic machinery components has also added to the notion of an absolute necessity for apoptosis during CFD. This is apodictic when, for instance, looking at abnormal CFD due to the loss of the p53/63/73 family transcription factors (Pan and Griep, 1995; Thomason et al., 2008, 2010; Van Nostrand et al., 2014, 2017; Richardson et al., 2017; Lin-Shiao et al., 2019). Second, it has become clear that the phenotypic investigative scope typically utilized to assess change has often been set low in magnification. Subtle changes in CFD such as in the size and shape of individual skeletal or dental elements — changes that by definition are not readily discernible — are likely to have been overlooked or undetected in many knock-out studies. However, when higher resolution examinations of specific biological systems are conducted, such as with the olfactory system and the loss of Caspases 3 and 7, detection of the smaller phenotypic alterations in CFD become evident (Cowan and Roskams, 2004).

### Apoptosis as Chisel II: Apoptosis in Abnormal Craniofacial Development

Analysis of both naturally occurring and targeted mutations in vertebrates has frequently included correlations between changes in the spatiotemporal topography of apoptosis and the larger phenotypic outcome of the mutation. Assessing the immense breadth of all such analyses lies well beyond our here intended scope; rather, to facilitate the notion of craniofacial apoptosis as chisel rather than sledge-hammer, we highlight four instances for which alteration of apoptosis has been invoked as integral to the phenotypic outcome of a mutation associated with craniofacially expressed genes. To aid the discussion, and to underscore the rationale of the hypotheses at the core of our second aim, we focus our examples around apoptosis and the molecular dynamics of jaw patterning. Specifically, we discuss (1) how the absence of *Satb2* leads to increased apoptosis within the CNC of the coordinated jaw developmental modules in which it is expressed, (2) the role of *Pbx* genes in controlling apoptosis in the nasal fin at λ and the plausible significance of this for CFD and evolution, (3) how attenuation of *Fgf8* expression leads to increased apoptosis in the CNC at the hinge as well as decouples jaw integration, and (4) how loss of *Foxg1* leads to drastically altered apoptotic topography in the SCE and its signaling centres that results in the loss of suppression of lower jaw molecular identity in the upper jaw primordia.

#### *Satb2*, Apoptosis, and the Elaboration of Jaw Modules

**FIGURE 3:**
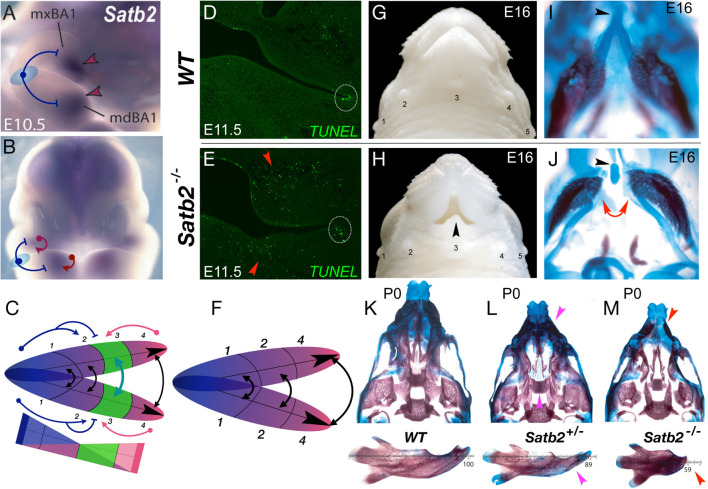
*Satb2*, apoptosis, and the elaboration of jaw modules. **(A,B)** Lateral **(A)** and frontal **(B)** aspects of E10.5 mouse embryos showing *Satb2* expression in BA1 and the FNP. Blue T-bars suggest a plausible hinge-related repression of *Satb2* and a red arrows a caps-related induction. **(C)** Schema depicting the coordinated nature of the *Satb2-*positive module (#3, in green). **(D,E)** Module restricted increase in apoptosis due to the loss of *Satb2*. Frontal section of wild type (WT) and *Satb2* null E11.5 embryos. Dotted white circle indicate the usual apoptosis at the oral commissure. **(F)** Schema of jaw development after the loss of the *Satb2*-positive module. **(G,H)** Gross anatomy of the snout of wild type (WT) and *Satb2* null embryos at E16. Black arrowhead indicates the maintenance of structure at the midline. 1–5, sinus follicles, noted for orientation. **(I,J)** Differential staining of bone (red) and cartilage (blue) of the samples in G and H. Red arrows indicate the drastic perisagittal loss of structure within Meckel’s cartilage of the *Satb2* module. Black arrowheads indicate the rostral process of Meckel’s cartilage, a structure developed at the midline. **(K–M)** Differential skeletal staining of P0 wild type, *Satb2* heterozygous and *Satb2* homozygous neonatal crania (top) and dissected mandibles (bottom) demonstrating the conspicuous nature of gene dosage on the elaboration of development of the *Satb2* module. Magenta arrowheads highlight deficiencies in *Satb2* heterozygotes, while red arrowheads highlight the greater losses in the same structures in the *Satb2* null skull.

As described earlier, reflective of the polarity in the developing jaw anlage and driven by the topography of its patterning centres, the jaw primordia are partitioned into distinct developmental modules (Figures 1L, 3C). Such modules are evolutionarily dissociable units consisting of integrated character states that are relatively independent of other nearby character states. Developmental regulation of such modules and their integration, including through control of intra-module cell population size, provides a key mechanism bridging CFD and evolution (Fish et al., 2011). Within the developing craniofacial primordia of late pharyngula stage embryos (and being most conspicuous from E10 onwards in mice), a *Satb2* (Special AT-rich sequence-binding protein 2) positive CNC population defines an integrated set of coordinated modules, topographically peri-sagittal to the midline, between the upper and lower jaws (Figure 3; Britanova et al., 2006; Fish et al., 2011). *Satb2* is a highly conserved member of a small, novel gene family that functions as a transcription factor which binds to nuclear matrix-attachment regions where it can simultaneously regulate transcription of multiple genes and augment the potential for enhancers to act over large distances (Britanova et al., 2006; Zarate et al., 2015). Satb2 is also a target for SUMOylation, a reversible modification of the protein that modulates its activity as a transcription factor.

Although originally identified as the human gene at *2q32-q33* associated with CP, it is now recognized as the core of *SATB2*-associated syndrome (SAS), a condition characterized by distinct craniofacial abnormalities, developmental delay, and intellectual disability (Zarate and Fish, 2017). Notably, it remains one of the few identified genes wherein haploinsufficiency in both humans and mice (roughly 25% of heterozygotes in the latter) is significantly associated with isolated CP (Britanova et al., 2006; Zarate and Fish, 2017). *Satb2*-dosage sensitivity in CFD is particularly conspicuous: Loss of both alleles of *Satb2* in mice leads to the morphogenic failure of the entire developmental module in which it is expressed, while leaving structures to either side autonomously developing (Figures 3G–J; Britanova et al., 2006). Mirroring the human condition, haploinsufficiency of *Satb2* in mice leads to hypoplasia of structures specifically derived from this module (Figures 3K–M). Significantly, in the absence of *Satb2*, high levels of apoptotic cells are found specifically within the mesenchyme of the effected modules (Figures 3D,E), providing a mechanistic link between a discrete module and the elaboration of its development and morphogeneis. Arguably, the relatively precise restriction of apoptosis and structural loss to the *Satb2*^+^ coordinated developmental modules themselves is mechanistically more chisel-like than sledgehammer-like in nature, and provides a framework to approach functional jaw integration and evolution (Fish et al., 2011).

#### *Pbx*, Apoptosis, and the Vertebrate Evolution of λ

**FIGURE 4:**
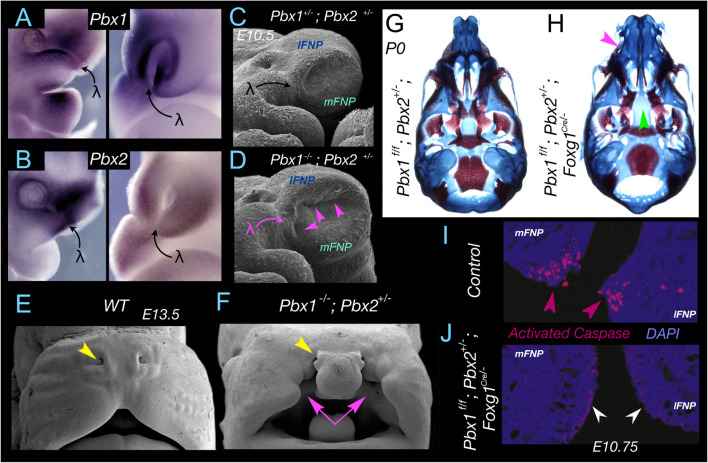
*Pbx*, apoptosis, and apical-apical epithelial apposition at λ. **(A,B)** *Pbx1* **(A)** and *Pbx2* **(B)** expression at λ in E10.75-E11 mouse embryos. Expression is found in both the epithelium and the CNC. **(C,D)** Significant alteration of λ and the topography of the mFNP, lFNP and mxBA1 appositions (magenta arrowheads) in *Pbx1*^–/–^; *Pbx2*^+/–^ mouse embryos. **(E,F)** Cleft lip and primary palate (magenta arrows) in a *Pbx1*^–/–^; *Pbx2*^+/–^ mouse embryo. Yellow arrowhead indicates the position of the external nares to deficiencies elsewhere (this compound mutant dies pre-term). **(G,H)** Demonstration of the role of *Pbx* in the epithelia of λ. Differential staining of bone (red) and cartilage (blue) of neonates in which Pbx1 has been flowed by a *Foxg1*^*Cre*^ driver that is expressed only in the SCE epithelia and not the CNC. **(G)** *Pbx1*^f/f^; *Pbx2*^+/–^ and **(H)** *Pbx*1^f/f^; *Pbx*2^+/–^; *Foxg1*^Cre/–^ I. Magenta arrow indicates the clefting of the primary palate. Green arrow indicates the clefting of the secondary palate. **(I,J)** Demonstration, through immunohistochemical detection of activated Caspase 3, of normal apoptosis at the nasal fin in a control embryo **(I)** and loss of apoptosis in the altered mFNP-lFNP apposition in *Pbx1*^f/f^; *Pbx2*^+/–;^ *Foxg1*^Cre/–^ embryos **(J)** at E10.75.

*Pbx* genes encode TALE (three amino acid loop extension) homeodomain transcription factors known for their interactions with *Hox* and *Meis* encoded proteins, and they govern disparate developmental processes including apoptosis in the seam of the nasal fin at λ (Selleri et al., 2001; Ferretti et al., 2011; Losa et al., 2018). Notably, *Pbx* genes display distinct expression patterns at λ in both the SCE and the subjacent CNC (Figures 4A,B). Compound mutations of *Pbx1* homozygosity and either *Pbx2* or *Pbx3* heterozygosity lead to craniofacial dysmorpholgies, including CL and CP, that are presaged by deficiencies during the development of λ (Figures 4C–F; Ferretti et al., 2011). Moreover, conditional inactivation of *Pbx1* specifically in the SCE (using a *Foxg1*^Cre^ driver) on a *Pbx2* or *Pbx3* heterozygous background likewise leads to CL and 1CP (Figures 4G,H) while CNC-specific inactivation using a *Wnt1*^Cre^ driver does not (Ferretti et al., 2011). Developmentally preceding CL and 1CP, combinatorial inactivation results in the arrest of the normal apoptosis at the seam of the nasal fin (Figures 4I,J).

Intensive investigation of *Pbx* regulatory control of development at λ has revealed an unique *Pbx-Wnt-p63-Irf6* regulatory module that correlates with the gradual increase in complexity at λ of the mxBA1–FNP connections and associated derived structures (e.g., the choanae, upper lips, and secondary palate) through evolutionary transitions among amniotes (Ferretti et al., 2011). Compound deficits of *Pbx* lead to decreased Wnt signaling at λ. Pbx and partner proteins bind to a *Wnt9/Wnt3* responsive element and drive expression at λ; notably, loss of *Wnt9b* also leads to CL and CP. *p63* is known for its transcriptional regulation of apoptotic activity (Yang et al., 1999; Gressner et al., 2005; Rinne et al., 2007; Pietsch et al., 2008; Dudgeon et al., 2009) and its deficiency in either human or mouse results in various craniofacial abnormalities, including CL and CP (Rinne et al., 2007; Thomason et al., 2008). Wnt signalling is mediated by Lef1/Tcf transcriptional binding to target genes, and it has been demonstrated that these Wnt signaling effectors transactivate *p63* through a specific regulatory region that is conserved within amniotes. Loss of either *Pbx* or *Wnt9b* leads to diminished p63 expression at λ and therefore loss of its contributions to the pro-apoptotic/anti-apoptotic balance. Furthermore, *Irf6*, the human homologue of which is the gene most commonly mutated in non-syndromic CL and CP (Dixon et al., 2011), is expressed in the MES and nasal fin seam epithelia; p63, in turn, directly binds to a known orofacial enhancer within the *Irf6* locus (Thomason et al., 2010). Unsurprisingly, *Irf6* is also down regulated at λ in both *Pbx* compound and *Wnt9b^–/–^* mutant embryos. Although the genomic elements regulating *p63* and *Irf6* within the Pbx-controlled module are conserved in mammals, they do not exist in other amniotes, Xenopus, or fish (Ferretti et al., 2011), a fact important to study of the biology and evolution of the mammalian λ. A mature λ is absent in fish and basal tetrapods and its complexity progressively grows within amniotes and mammals, where in the latter it is most developmentally and functionally complex and critical for the morphogenesis and positioning of, among other things, the choanae, upper lips, and primary and secondary palates (Compagnucci et al., 2011). It is conceivable that the *Pbx*-instigated regulatory module at λ is an evolutionary innovation associated with the appearance in mammals of an elaborated choanae and secondary palate. Investigations of these molecular dynamics in crocodilians, which through a processes of convergent evolution also possess secondary palates, have yet to be conducted but will likely be of note. Overall, the increased complexity of the regulatory module itself correlates with the gradual increase in complexity of λ and associated structures through evolutionary transitions from basal tetrapods to amniotes and then to mammals. Notably, at the heart of the module is the effectuation of apoptosis at λ, a notion further confirmed by the rescue of CL and CP by the reintroduction of apoptosis at the nasal fin in the *Pbx* compound mutants wherein the *Wnt* signaling cascade is conditionally activated in the same epithelial cells in which *Pbx* has been conditionally inactivated (Ferretti et al., 2011; Losa et al., 2018). It would seem, then, that significant evolutionary advances have followed an increasingly involved genomic blueprint for the precise, scalpel-like apoptotic sculpting of λ.

#### *Fgf8*, Apoptosis, and Developmental Integration at the Hinge

**FIGURE 5:**
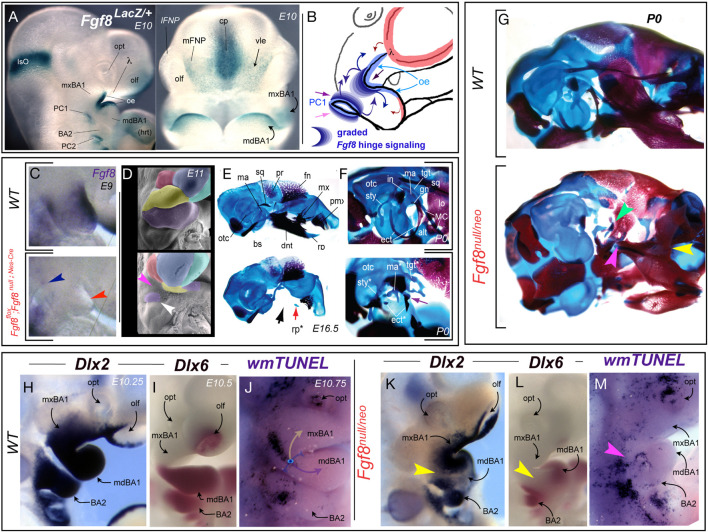
*Fgf8*, apoptosis, and developmental integration at the hinge. **(A)** *Fgf8* expression, notable in the oral ectoderm (oe) and pharyngeal cleft (PC1) as assayed by X-gal staining due to β-galactosidase activity resulting from the insertion of a *LacZ* cassette in the *Fgf8* locus. **(B)** Schema depicting the graded *Fgf8* signaling environment that the BA1 CNC must interpret at the hinge. **(C–F)** Tissue specific inactivation of *Fgf8* in the oral ectoderm and pharyngeal cleft. **(C)** *Fgf8* expression at E9 demonstrating (red arrowhead) that the gene has been inactivated in the oral ectoderm of BA1. Blue arrowhead, slight persistent PC1 expression. **(D)** Psuedocoloured scanning electron micrographs of wild type and *Fgf8*^flox^; *Fgf8*^null;Nes–Cre^ E11 embryos. White arrowhead, mdBA1 tissue associated with the developing malleus. Magenta arrowhead, catastrophic loss of most of the BA1 primordia. **(E–G)** Differential staining of bone (red) and cartilage (blue) in E16 (E) and P0 (F) wild type and *Fgf8*^flox^; *Fgf8*^null;Nes–Cre^ embryos. Black arrow, devastating loss of hinge-associated structure. Red arrow, remnants of the mdBA1 cap associated rostral process (rp). Purple arrow, the remnants of the ectotympanic (etc) and malleus (ma). **(G)** Genetic attenuation of *Fgf8* demonstrating the disintegration of hinge structures. Yellow arrowhead, dentary-maxillary synganthia. Magenta arrowhead, loss of the lower jaw articulation structures. Green, malleo-ectotympanic tissue. **(H–M)** Topography of altered gene expression and apoptosis in E10.25–10.75 mouse embryos. **(H,K)** *Dlx2* expression. Yellow arrowhead in K, separation in oral and aboral domains due to decreased *Fgf8* levels across the huge region. **(I,L)** *Dlx6* expression. Yellow arrowhead in L, separation in oral and aboral domains due to decreased *Fgf8* levels. **(J,M)** Alterations of apoptotic topography, including loss of normal mxBA1 apoptotic swath (magenta arrowhead).

*Fgf8* encodes a potent vertebrate morphogen that is dynamically expressed during SCE ontogeny and its loss leads to increased apoptosis in the hinge region CNC and changes in the molecular topography of the SCE (Crossley and Martin, 1995; Trumpp et al., 1999; Griffin et al., 2013). Cumulative evidence from many experimental sources and paradigms indicates that the elaborate ontogeny of *Fgf8* expression in the SCE reflects a dynamic and significant signaling environment to be encountered by the CNC responsible for generating rostral cranial skeletal structures (Trumpp et al., 1999; Abu-Issa et al., 2002; Frank et al., 2002; Storm et al., 2006; Depew and Compagnucci, 2008; Compagnucci et al., 2011; Griffin et al., 2013). In addition to being an organizing factor at the anterior neural ridge and the isthmus (midbrain - hindbrain boundary), *Fgf8* is expressed at the hinge in PPt1 epithelia and the hinge-centric OE of mxBA1 and mdBA1 (Figures 5A,B). Thus, the hinge as an organizing centre involves the coordinated integration two local sources of Fgf8.

Utilizing a tissue specific, *Nestin*^Cre^-mediated BA1 conditional knockout of *Fgf8* it has been demonstrate that loss of *Fgf8* in the OE (including BA1) of the mouse is accompanied by the catastrophic loss of the hinge region of both the upper and lower jaws. This included of all the developmental modules within BA1 except those furthest from the hinge and centred at the caps (see *Fgf8^flox^; Fgf8^null;Nes–Cre^* in Figures 5C–F; Trumpp et al., 1999). The presence of some caps signaling is reflected, for instance, by the retention of the midline rostral process of Meckel’s cartilage (mdBA1 cap) and the well developed premaxillae (λ cap; Figures 5E,F). In these experiments the inactivation of *Fgf8* in the PPt1 notably occurred slightly later and was correlated with the maintenance of a small population of cells in BA1 at the PC1 (Figure 5D) that gave rise to the malleus (the mammalian articular element homologue) and portions of the ectotympanic (Figures 5E,F). Cell proliferation assays of BrdU incorporation, combined with Nile blue sulfate and TUNEL assays, demonstrated that the patent catastrophic losses in the mutants were due to extensive apoptosis of the BA1 CNC (Trumpp et al., 1999). This established Fgf8 as an essential signaling factor of the vertebrate BA1 hinge and indicated that expression in the OE and PPt is both integral to the normal formation of the jaw skeleton.

Among the approaches subsequently used address the relationship between levels of Fgf8 signaling and the complex craniofacial skeleton has been the employment of genetic attenuation of *Fgf8* dosage in mice through use of combinations of hypomorphic (*Neo*) and null murine *Fgf8* alleles. This allows for a modulation of Fgf8 signaling by reducing functional expression levels to approximately 20% (*Fgf8*^null/Neo^), 40% (*Fgf8*^Neo/Neo^), 50% (*Fgf8^+/null^*^)^, or 70% (*Fgf8^+/Neo^*) of normal (wild-type) levels (Meyers et al., 1998; Abu-Issa et al., 2002; Frank et al., 2002; Storm et al., 2003; Storm et al., 2006; Griffin et al., 2013). These graded challenges to *Fgf8*-regulated cranial morphogenesis demonstrate that *Fgf8* dosage determines murine mid-facial integration and polarity within the nasal and optic capsules (Griffin et al., 2013). The highest levels of attenuation lead to disruption of the normal morphological continuity of jaw and associated skeletal structures (Figure 5G). In *Fgf8*^null/Neo^ mutants, syngnathia develops between the distal dentary and the nasal-capsule associated portions of the maxilla. Specifically, this involves loss of the components of both the functional lower jaw articulation (i.e., the proximal dentary structures such as the condylar, coronoid, and angular processes) and the functional upper jaw articulation (e.g., articular portion of the squamosal; Figure 5G). Skeletal structures associated with the PPt1 (e.g., malleus, ectotympanic, goniale, incus, and tegmen tympani) are truncated and dysmorphic while BA2 derivatives (e.g., stapes and Reichert’s cartilage) are either lacking or highly dysmorphic. Attenuation of Fgf8 signal strength thus challenges integration between the OE and PPt signals, a point further supported by the loss of normal continuity through the oral-aboral axis of BA1 of responsive craniofacial regulatory genes such as *Dlx2* and *Dlx6* (Figures 5H,I,K,L). Notably, these changes correlate with a significant alteration in the regional topography of apoptosis (Figures 5J,M).

#### *Foxg1*, Apoptosis, and Control of Upper Jaw Patterning and Development

*Foxg1* is an evolutionarily conserved member of the *Fox/Forkhead* family of winged-helix transcription factors (Xuan et al., 1995). *Foxg1* has principally been investigated for its crucial role in forebrain development; however, its loss has recently been shown to also affect upper jaw patterning and the topographic profiles of apoptosis in the SCE associated the hinge (Xuan et al., 1995; Hanashima et al., 2004; Danesin and Hobart, 2012; Hou et al., 2020; Compagnucci and Depew, submitted). With regard to its role in CNS development, *Foxg1* functions to regulate forebrain induction, regional patterning, and corticogenesis and mice lacking functional *Foxg1* have significant cephalic hypotrophy with substantial morphogenic deficits of telencephalic structures, including dorsally of the neocortex and ventrally of the basal ganglia. Sufficient evidence of neurodevelopmental deficits stemming from *FOXG1* haploinsufficiency or duplication in humans has led to the recognition of “FOXG1-related disorders” as a distinct clinical classification variously involving microcephaly, mental impairment, autism spectrum disorders, and a congenital variant of Rett (RTT) syndrome (Ariani et al., 2008; Florian et al., 2012; Mitter et al., 2018; Hou et al., 2020). Importantly, FOXG1 syndrome in humans is also typified by characteristic craniofacial features, including a prominent metopic suture, large ears, bilateral epicanthic folds, a bulbous nasal tip, depressed nasal bridge, tented or thickened upper lip, prognathism, hypermetropia, and synophyrys (Florian et al., 2012). A significant linkage between the *FOXG1* locus at 14q12 and non-syndromic orofacial clefting has also recently been reported (Mohamad Shah et al., 2018).

**FIGURE 6:**
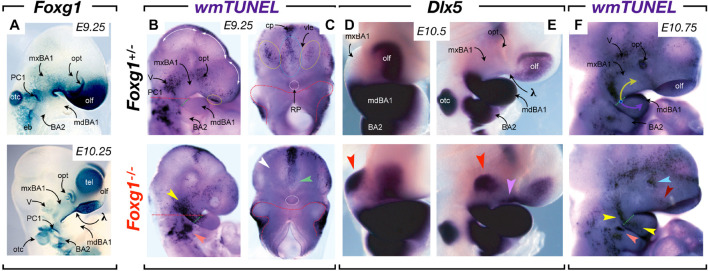
*Foxg1*, apoptosis, and control of upper jaw patterning and development. **(A)** *Foxg1* expression, notable in mxBA1, FNP and hinge SCE, and absence in mdBA1, as assayed by X-gal staining due to β-galactosidase activity resulting from the insertion of a *LacZ* cassette in the *Foxg1* locus. **(B,C)** Whole mount TUNEL assay of E9.25 *Foxg1^+/–^* and *Foxg1^–/–^* embryos. **(B)** Lateral view. Dotted red lines indicate planes of separation for **(C)**. Dotted green lines indicate the nascent hinge region. Dotted yellow circles indicate the normal foci of apoptotic cells in the medial olfactory fields of the SCE. Yellow arrowhead, extensive ectopic apoptosis connecting the trigeminal, the aboral border of mxBA1, and the hinge (including the oral ectoderm) evinced in null embryos. White arrowhead, loss of apoptotic foci in the medial olfactory fields of the SCE. Green arrowhead, loss in the ventral SEC below the closed anterior neuropore. **(D,E)** Frontal **(D)** and lateral **(E)** views of E10 *Foxg1^+/–^* and *Foxg1^–/–^* embryos highlighting the ectopic induction of *Dlx5* (red arrowhead). A master regulatory gene for mdBA1 identity, expression in the mxBA1 demonstrates that Foxg1 acts to suppress the acquisition of lower jaw identity in the upper jaw developmental field. Lavender arrowhead, ectopic epithelial expression of *Dlx5*. **(F)** Apoptosis in E10.75 *Foxg1^+/–^* and *Foxg1^–/–^* embryos. Yellow arrow, mxBA1 hinge signaling. Lavender arrow, mdBA1 hinge signaling. Dotted green line, oral-aboral axis at the hinge. Yellow arrowheads, alterations of mxBA1 apoptosis and expansion into mdBA1. Orange arrowhead, ectopic BA2 apoptotic foci. Light blue arrowhead, alteration of apoptosis in the lens pit. Dark red arrowhead, reorientation of apoptosis normally associated with the nasolacrimal groove.

Notably, *Foxg1* is broadly expressed in the SCE overlying the upper jaw primordia (including λ) and hinge (Figure 6A). Loss of function in mice reveals that *Foxg1* is required during CFD for both neurocranial and viscerocranial development, including the sensory capsules, neurocranial base, middle ear, and upper jaws (Compagnucci and Depew, submitted). Notably, *Foxg1* acts to suppress the induction of lower jaw identity in the upper jaw primordia as without *Foxg1* mxBA1 partially acquire deep mdBA1 molecular identities (exemplified by the ectopic expression of lower jaw master-regulatory gene, *Dlx5*; Figures 6D,E). Significantly, changes in middle ear and upper jaw development are preceded by drastic alterations of the topography of apoptotic PCD within the SCE, including the BA1 epithelia at the hinge and that between the frontonasal (facial) ectodermal zone and the olfactory placode (Figures 6B,C,F). For instance, at E9.25, the *Foxg1^+/–^* embryonic SCE exhibits the well characterized foci of apoptotic cells associated with the sculpting of the PPR (see above), as well as associated with the recently closed anterior neuropore (and commissural plate) and the medial olfactory field (i.e., just lateral to the *Fgf8+* ventrolateral epithelia). In *Foxg1^–/–^* littermates these fields are all altered: apoptosis is undetected in the medial olfactory field and it is expanded in the middle SCE of BA2. Most noticeably, extensive ectopic apoptosis is found along the aboral border of BA1 and bridging the OE and PC1 at the hinge (Figures 6B,C). A day and a half later in development and the usual topography of apoptotic cells in the SCE retains its predictable nature in *Foxg1^+/–^* embryos. This includes an elongate swath, essentially restricted to mxBA1, that runs dorsoventrally from the caudal end of the trigeminal ganglion and ends along the OE-PPt1 oral-aboral axis (Figure 6F). It also includes a patch centrally located on the SCE of BA2. In mutants, however, the mxBA1 apoptotic foci is restricted oro-aborally and extends from mxBA1 into mdBA1. Thus, with the loss of *Foxg1*, a change of apoptotic topography in the craniofacial primordia presages the eventual disruption of mxBA1 patterning and development. This is particularly notable as these changes occur where the hinge and upper cap signaling centres are being established.

### Apoptosis: A Scalpel for Craniofacial Development Patterning and Plausibly Unique Roles in CFD

Using the genetic perturbation of *Satb2*, *Pbx*, *Fgf8*, and *Foxg1* as exemplars, we have examined the correlation of aberrant apoptosis in the elaboration of jaw modules, the evolution and elaboration of λ, the developmental integration at the mandibular arch hinge, and the control of upper jaw identity, patterning and development. With each of these examples we have presented a correlation between a targeted mutation, changes in apoptotic profiles, and abnormal CFD with particular focus on abnormal jaw development. We have also seen that apoptosis is evident at stereotypic and predictable locations during normal CFD. Apoptotic cells, for example, are readily detectable in embryos in locations wherein epithelial-epithelial appositions are occurring (e.g., the palatal shelves, the nasal fin, the oronasal, and buccopharyngeal membranes) as well as within craiofacial epithelia that are undergoing obvious and significant morphogenesis (e.g., the lens pit and developing mammalian molars). Additionally, we have seen that normally observable focal patches of apoptotic cells correlate with the placodal compartmentalization within the SCE.

**FIGURE 7:**
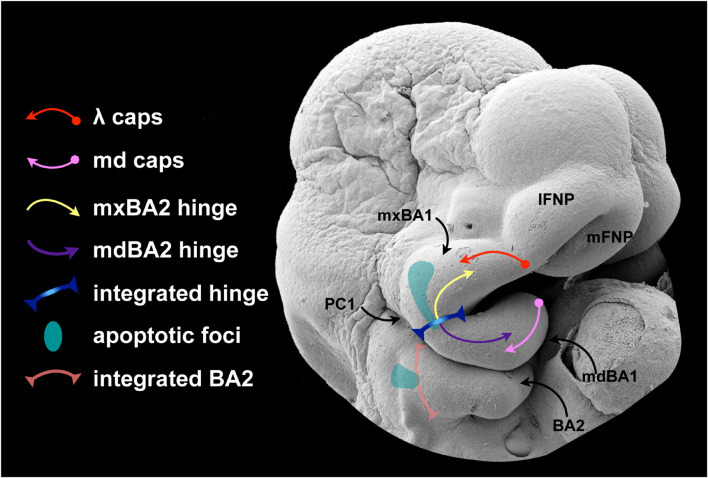
A proposed role for apoptosis in hinge and caps signaling.

Apoptotic cells are, however, also notably detectable during CFD in topographies that do not readily lend themselves either to an aspect of the epithelial morphogenesis or apposition or to structural compartmentalization. This is exemplified, for instance, by the patch of apoptotic cells found within mxBA1 ectoderm at E10.5 in mice (see Figures 5J, 6F). Moreover, in our primer on CFD we suggested that among the fundamental tasks in patterning BA1 and the jaws were the following: (1) to establish upper jaw versus lower jaw positional identity; (2) to generate and maintain the point of articulation between the upper and lower jaw arcades; and (3) to keep the jaw arcades in appropriate functional registration during subsequent development and morphogenesis. We posit here that the mxBA1 apoptotic patch facilitates the actualization of these tasks. More specifically, we posit that PCD in this location uniquely facilitates both the patterned integration of the two components of the hinge signal — the PPt1 and the OE — and the patterned suppression of lower jaw molecular identity form upper jaw cellular primordia (Figure 7).

It is patent that developmental mechanisms must be employed that properly instruct an equipotent, fungible population of CNC to (1) discriminate instructions for upper jaw pattern and development from those for lower jaw pattern and development and (2) then discriminate and integrate both OE-derived, *Fgf8*-generated developmental cues and aboral, PPt1-derived *Fgf8*-generated developmental cues. We know that *Foxg1*, restricted in its expression to the upper jaw-associated SCE as it is, is part of solution to the first task: in its absence, normal structural development across the hinge is lost and a large domain of mxBA ectopically acquires a mdBA1 molecular identity. Thus *Foxg1* makes the epithelial environment of the upper jaw distinct from that of the lower jaw. Included in this environment is control of the foci of apoptotic cells in the SCE. Though admittedly full understanding of the correlation of morphogen level and morphogenic outcome has yet to be achieved, we also know that thresholds of morphogens such as *Fgf8* are necessary for normal patterning across the hinge. Perturbation of morphogen levels leads to alteration of gene expression, morphology and the topography of apoptosis. These two things — control of epithelial environment and control of morphogen concentration — are likely linked on numerous levels.

Apoptotic control of a morphogen producing cell population is not an undocumented occurrence during development. Cell death has previously been proposed as a mechanism for regulating the effective potency of a secreted morphogen by regulating the numbers of cells producing the factor. Such a mechanism for patterning has been suggested for the PCD associated with Shh-producing cells in the limb bud, *Fgf8*-producing cells of the anterior neural ridge, and the epithelial patterning centres of the enamel knot of developing teeth (Vaahtokari et al., 1996; Sanz-Ezquerro and Tickle, 2000; Nonomura et al., 2013; Ahtiainen et al., 2016). In each of these cases, control of the patterning influence of the signaling molecule is through the apoptotic removal of the morphogen producing cells themselves.

With regard to the SCE, we suggest that another mechanism to regulate the effective elaboration of patterning signals is to program apoptotic foci as a means of micromanaging epithelial environments that inform the CNC. It is possible that the mxBA1 apoptotic foci, for example, acts to restrict the spread of a hinge signal that could otherwise easily be interpreted by regionally subjacent mxBA1 CNC to make them act as though they were mdBA1 CNC. Either physical presentation of factors from the apoptotic cells or the topographic restrictions of the effective spread of a signal through the epithelium could both subtly but effectively micromanage CNC interpretations of local patterning cues either specific to mxBA1-hinge signals or for integrated OE and PPt1 signaling (schmatized in Figure 7). In either case, it is conceivable that, rather than having no biological function, the predictable apoptotic foci apparent in mxBA1 acts as a subtle sculptor of patterning cues regulating important, though not necessarily life enabling, aspects of CFD and morphogenesis.

Similar roles may well be posited for other patches of apoptosis detected during CFD, including those found in the SCE between the anterior neuropore and the olfactory placode. However, while we have some regard for the topography of the normal apoptotic profile during CFD, understanding of the total picture of the spatiotemporal topography of apoptosis during vertebrate CFD — let alone all of its plausible functions — has yet to be fully realized. With regard to roles for apoptosis in normal CFD and patterning, moreover, we would suggest that investigative optics are better set at higher magnification and evidence of a scalpel’s touch rather than a sledgehammer’s punch be sought as we address the goal of understanding the roles that apoptosis plays during CFD.

## Author Contributions

MD and CC conceptualized the themes presented and prepared the manuscript. All authors participated in data collection and interpretation, contributed to the article, and approved the submitted version.

## Conflict of Interest

The authors declare that the research was conducted in the absence of any commercial or financial relationships that could be construed as a potential conflict of interest.

## Publisher’s Note

All claims expressed in this article are solely those of the authors and do not necessarily represent those of their affiliated organizations, or those of the publisher, the editors and the reviewers. Any product that may be evaluated in this article, or claim that may be made by its manufacturer, is not guaranteed or endorsed by the publisher.
